# Steatotic Liver Disease in Newly Diagnosed and Gluten-Free Diet-Treated Adults with Celiac Disease

**DOI:** 10.3390/nu18203419

**Published:** 2026-10-18

**Authors:** Daniel Vasile Balaban, Laura Ioana Coman, Iulia Enache, Mihnea Cezar Gabriel Oprea Toma, Valentin Andrei Raduinea, Alexandru Marius Stancu, Sandica Nicoleta Bucurica, Raluca Simona Costache, Alina Popp, Mariana Jinga

**Affiliations:** 1 Faculty of Medicine, Internal Medicine and Gastroenterology Discipline, “Carol Davila” University of Medicine and Pharmacy, 020021 Bucharest, Romania; vasile.balaban@umfcd.ro (D.V.B.); iulia.enache@drd.umfcd.ro (I.E.); valentin-andrei.raduinea0721@stud.umfcd.ro (V.A.R.); alexandru-marius.stancu0721@stud.umfcd.ro (A.M.S.); sandica.bucurica@umfcd.ro (S.N.B.); raluca.costache@umfcd.ro (R.S.C.); alina.popp@umfcd.ro (A.P.); mariana.jinga@umfcd.ro (M.J.); 2 Gastroenterology Department, “Dr. Carol Davila” Central Military University Emergency Hospital, 010825 Bucharest, Romania; mihnea.oprea@rez.umfcd.ro; 3 Medical Center for Diagnosis, Outpatient Treatment and Preventive Medicine, 011794 Bucharest, Romania; 4 National Institute for Mother and Child Health “Alessandrescu-Rusescu”, 020382 Bucharest, Romania

**Keywords:** celiac disease, gluten-free diet, fatty liver, steatosis, controlled attenuation parameter

## Abstract

*Background and Aims:* Celiac disease (CD) is increasingly recognized as a driver of metabolic alterations and liver dysfunction, also after initiation of the gluten-free diet (GFD). Our aim was to evaluate the metabolic profile and hepatic steatosis prevalence in patients with CD at diagnosis compared with those on long-term GFD. *Methods:* We conducted an observational cross-sectional study with an exploratory longitudinal analysis, including adult CD patients evaluated either at diagnosis (CD_new group) or during follow-up after at least 24 months of self-reported adherence to GFD (CD_GFD group); a non-CD patient group, who underwent the same diagnostic pathway but in whom CD was excluded, was used as control. Patients with diabetes and other chronic liver diseases were excluded. Clinical, metabolic, and hepatic parameters were assessed, including lipid profile, fasting glucose, and liver steatosis, determined by abdominal ultrasound and transient elastography with controlled attenuation parameter (CAP). Steatosis was defined as CAP ≥ 248 dB/m. *Results:* A total of 85 patients were included (27 CD_new, 40 CD_GFD, 18 non-CD controls). Patients in the CD_GFD group had higher body mass index compared with CD_new (24.5 vs. 21.9 kg/m^2^), while age and sex distribution were similar. Total cholesterol was numerically higher in CD_GFD (183.1 vs. 172.9 mg/dL), whereas triglycerides, HDL-C, and LDL-C were comparable between groups. Fasting glucose levels were similar and within the normoglycemic range. Hepatic steatosis was numerically more prevalent in CD_GFD, with ultrasound-defined steatosis observed in 37.5% compared to 25.9% in CD_new and 33.3% in non-CD controls without reaching statistical significance. CAP-defined steatosis was present in 32.5% of CD_GFD patients versus 18.5% in CD_new and 33.3% in non-CD controls, with numerically higher mean CAP values in the follow-up group (217 vs. 204 dB/m). In the exploratory longitudinal subset (*n* = 10), median CAP increased from 182.5 to 217.0 dB/m (*p* = 0.047). Sensitivity analysis using higher CAP thresholds showed that higher-grade steatosis was observed more frequently in GFD-treated patients. Liver stiffness measurements were similar between groups (4.3, 4.5, and 4.9 kPa, respectively). *Conclusions:* Long-term GFD-treated CD patients had higher BMI and numerically higher hepatic steatosis prevalence, although steatosis differences were not statistically significant and GFD-treated status was not independently associated with CAP-defined steatosis after adjustment. CD patients may present distinct metabolic profiles from diagnosis to long-term follow-up.

## 1. Introduction

Celiac disease (CD) is a chronic immune-mediated enteropathy with systemic features, triggered by gluten ingestion in genetically predisposed individuals [1]. With a prevalence of about 1%, CD is one of the most common chronic digestive diseases worldwide [1,2].

Beyond intestinal pathology and its nutritional consequences, along with bowel cancer risk in the long term, increasing attention has been recently directed towards the systemic metabolic disturbances in CD. While untreated CD is usually associated with alterations in lipid metabolism due to fat malabsorption, a high prevalence of metabolic disturbances has also been reported in long-term gluten-free diet (GFD)-treated CD patients.

GFD is the mainstay of treatment in CD, essential for mucosal healing and reducing complications [3], but an unbalanced diet can result in nutritional disorders, weight gain, and the occurrence of metabolic syndrome [4,5]. In this setting, metabolic dysfunction-associated steatotic liver disease (MASLD), the most common chronic liver disease worldwide [6], usually occurring in individuals at metabolic risk, has also become an emerging comorbidity in CD [4,7]. Liver steatosis has been reported both at CD diagnosis and after GFD introduction, with mechanisms involving altered lipid absorption, disruption of the gut–liver, axis and consumption of processed gluten-free foods that are rich in simple carbohydrates [8,9].

While some studies have compared metabolic profiles between newly diagnosed and GFD-treated CD patients using standard biochemical and imaging techniques, the use of controlled attenuation parameter (CAP) measured by transient elastography [10] has not been extensively applied in CD cohorts [11].

In the present study, we primarily aimed to compare metabolic characteristics and imaging-defined hepatic steatosis across newly diagnosed CD versus long-term GFD-treated CD patients. In addition, in a subset of patients with paired diagnostic and follow-up assessments, we explored within-person changes during GFD follow-up.

## 2. Methodology

### 2.1. Study Design and Participants

We conducted a single-center retrospective observational study with a primary cross-sectional component and an exploratory partial longitudinal analysis, including adult patients evaluated in our gastroenterology clinic between 2019 and 2025. The clinical data analysed in this study were collected during routine patient care. At admission, patients provided informed consent for the use of their medical data for research purposes. All eligible patients with the required routine-care assessments available during the study period were included. Three groups were analysed: patients with newly diagnosed CD assessed at the time of diagnosis (CD_new), patients with established CD evaluated after at least 24 months of self-reported adherence to gluten-free diet (CD_GFD), and non-CD controls. The control group consisted of patients referred for suspected CD (based on clinical presentation or suggestive routine laboratory work-up) who underwent the same diagnostic pathway, including serological and histological assessment, but in whom CD was ultimately excluded; this clinical pathway for referral is subjected to selection bias. The same clinical, metabolic, ultrasonographic, and transient elastography assessment protocol was applied across all study groups. We excluded patients with ongoing or suspected pregnancy, associated chronic liver diseases (except MASLD), use of potentially steatogenic drugs, chronic alcohol intake, type 1/2 diabetes mellitus, and those taking lipid-lowering or glucose-lowering therapies.

In addition to the primary cross-sectional analysis, an exploratory longitudinal analysis was performed in patients for whom paired assessments were available both at the time of CD diagnosis and after at least 24 months of GFD. This subgroup comprised all patients from the study cohort who fulfilled this paired-data availability criterion. Within-patient changes in metabolic parameters, hepatic steatosis assessed by ultrasound and CAP, and liver stiffness were subsequently evaluated between diagnosis and follow-up.

### 2.2. Diagnostic Assessment

CD was diagnosed according to the 2019 European Society for the Study of Coeliac Disease (ESsCD) guideline, based on standard serologic and histologic criteria [12]. Serological testing included dosing of anti-tissue transglutaminase IgA (anti-tTG IgA, cut-off for positivity of 10U), together with total serum IgA; in patients with IgA deficiency, anti-tTG IgG antibodies were further measured. Duodenal histology was obtained using a standardized biopsy protocol of four distal duodenal biopsies and one-to-two bulb biopsies. Histopathological assessment of biopsy specimens was performed according to the Marsh–Oberhuber classification. All newly diagnosed CD patients were biopsy-proven CD, with mucosal atrophy on duodenal histology (Marsh ≥ 3).

GFD adherence was based on patient self-reporting during clinical follow-up and was additionally characterized by follow-up celiac serology and repeat duodenal histology in all GFD-treated patients.

### 2.3. Clinical, Metabolic, and Liver Assessment

Demographic and anthropometric parameters included age, sex, and body mass index (BMI). BMI was calculated as body weight divided by squared height (kg/m^2^). Blood samples were obtained in the fasting state for measurement of fasting glucose and lipid parameters. Liver enzymes were not included as study outcomes because hypertransaminasemia in untreated CD may reflect celiac-associated hepatitis and frequently improves following GFD; in this setting, aminotransferase elevations cannot be reliably attributed to imaging-defined SLD. Hepatic steatosis was assessed using two complementary imaging techniques: conventional abdominal ultrasonography and vibration-controlled transient elastography with controlled attenuation parameter (CAP) [13]. Ultrasound examinations were performed by a single experienced examiner, not blinded to the clinical status of the patient. Liver steatosis was defined qualitatively according to increased hepatic echogenicity relative to the renal cortex, posterior beam attenuation, and impaired visualization of intrahepatic structures [14,15]. Transient elastography was performed in a fasting state by two experienced non-blinded operators using FibroScan (Echosens, Paris, France) and following the same examination protocol. The M probe was used as standard, while the XL probe was used in patients with BMI ≥ 30 kg/m^2^. A technically reliable examination required at least 10 valid measurements, a success rate ≥ 60%, and an IQR/median ratio ≤ 0.30 [16]. CAP was expressed in dB/m and liver stiffness in kPa. The prespecified threshold for the primary definition of hepatic steatosis was CAP ≥ 248 dB/m. Because published CAP thresholds vary according to the desired degree of steatosis and study population, sensitivity analyses were additionally performed using thresholds of ≥268 and ≥285 dB/m [17,18].

### 2.4. Study Outcomes

The primary outcome was the prevalence of liver steatosis in the two main groups: newly diagnosed and GFD-treated CD patients, assessed independently by ultrasound and CAP. Secondary outcomes included differences in BMI, fasting glucose, lipid profile, mean CAP value, and liver stiffness between groups. Additional analyses compared hepatic steatosis parameters with those observed in non-CD controls and evaluated the robustness of CAP-defined steatosis prevalence using alternative CAP thresholds. To explore whether GFD-treatment status was independently associated with hepatic steatosis, additional multivariable analyses were performed among patients with CD. CAP-defined steatosis (≥248 dB/m) was evaluated using multivariable logistic regression, with CD group (GFD-treated versus newly diagnosed), age, sex, and BMI entered as covariates.

### 2.5. Statistical Analysis

Statistical Analysis was carried out using Microsoft Excel and SPSS Statistics 31. Continuous variables are reported as mean ± SD or median, while categorical nominal variables are presented as proportion (%). Distribution patterns were assessed using the Shapiro–Wilk test and visual inspection. Normally distributed continuous variables were reported as mean ± SD and compared using t-tests/ANOVA, whereas non-normally distributed variables were reported as median (IQR) and compared using Mann–Whitney or Kruskal–Wallis tests. Categorical variables were analysed using the chi-square test or Fisher’s exact test, as appropriate. All tests were two-sided, and *p* < 0.05 was considered statistically significant. Within-patient continuous changes were evaluated using the Wilcoxon signed-rank test and paired categorical outcomes using the exact McNemar test. Given the exploratory nature of the study, which was primarily intended to estimate the magnitude of between-group differences and generate hypotheses for larger prospective studies, no formal a priori sample-size calculation was performed.

## 3. Results

Altogether, 85 participants were included: 27 newly diagnosed CD patients (CD_new), 40 GFD-treated CD patients (CD_GFD), and 18 non-CD controls. Median age was 40 years in CD_new and 42 years in CD_GFD (*p* = 0.578), with women representing 88.9% and 80.0% of the groups, respectively (*p* = 0.504). GFD-treated patients evaluated at follow-up had a significantly higher BMI than newly diagnosed patients (24.5 vs. 21.9 kg/m^2^, *p* = 0.035), while mean BMI in non-CD controls was 23.1 kg/m^2^-Table 1. Histological assessment revealed atrophic mucosal injury in all newly diagnosed CD patients, while at follow-up, there were four patients with persistent mucosal atrophy.

Concerning lipid profile, total cholesterol was numerically higher in CD_GFD than in CD_new (183.1 ± 42.9 vs. 172.9 ± 43.5 mg/dL, *p* = 0.358), but no significant between-group differences were observed for HDL-C (60.1 ± 17.4 vs. 57.3 ± 17.7 mg/dL, *p* = 0.637) or LDL-C (107.1 ± 36.8 vs. 109.5 ± 35.7 mg/dL, *p* = 0.830). Also, similar triglyceride concentrations were seen among the two CD groups (79.8 ± 32.5 in CD_GFD vs. 84.1 ± 32.5 mg/dL in CD_new, *p* = 0.605); see Table 2.

Fasting glucose concentrations were comparable across the three groups (86.9 ± 12.6 mg/dL in CD_new, 88.6 ± 10.0 mg/dL in CD_GFD, and 87.3 ± 10.5 mg/dL in non-CD controls), indicating average normoglycemia in all cohorts.

Hepatic steatosis was identified in 25.9% (95% CI 13.2–44.7%) of CD_new patients by ultrasound and in 18.5% (95% CI 8.2–36.7%) by CAP, whereas in the CD_GFD group, prevalence rates were 37.5% (95% CI 24.2–53.0%) and 32.5% (95% CI 20.1–48.0%), respectively. In direct comparisons between the two CD groups, ultrasound-defined steatosis was numerically more frequent in CD_GFD than CD_new patients (prevalence ratio 1.45, 95% CI 0.68–3.07; Fisher’s exact *p* = 0.428). Similarly, CAP-defined steatosis was numerically more frequent in CD_GFD patients (PR 1.76, 95% CI 0.71–4.35; *p* = 0.266). Mean CAP values were numerically higher in the CD follow-up group compared to newly diagnosed patients (217.0 vs. 204.1 dB/m), consistent with a greater burden of hepatic steatosis. Both ultrasound-defined and CAP-defined hepatic steatosis were observed in similar proportions in the CD_GFD group as compared to non-CD controls (Table 3). Using CAP ≥ 248 dB/m as the binary CAP definition, ultrasound and CAP classifications were concordant in 75/85 assessments (88.2%), with a Cohen’s κ of 0.724 (95% CI 0.565–0.883), indicating substantial agreement.

In exploratory multivariable analysis, GFD-treated status was not independently associated with CAP-defined hepatic steatosis after adjustment for age, sex, and BMI (aOR 0.69, 95% CI 0.15–3.15; *p* = 0.629). In contrast, higher BMI was independently associated with greater odds of CAP-defined steatosis (aOR 1.28 per 1 kg/m^2^ increase, 95% CI 1.06–1.55; *p* = 0.010). Age and female sex were not significantly associated with the CAP outcome.

Sensitivity analyses using higher CAP thresholds showed a greater numerical separation between newly diagnosed and GFD-treated CD patients, although the overall comparisons remained statistically nonsignificant. CAP ≥ 268 dB/m was observed in 1/27 (3.7%) CD_new patients compared with 10/40 (25.0%) CD_GFD patients and 4/18 (22.2%) non-CD controls (*p* = 0.065). At the CAP ≥ 285 dB/m threshold, no newly diagnosed patient fulfilled criteria for steatosis, compared with 5/40 (12.5%) GFD-treated patients and 3/18 (16.7%) non-CD controls (*p* = 0.075). Thus, higher-grade CAP-defined steatosis was uncommon at CD diagnosis but more frequently observed in both GFD-treated CD patients and non-CD controls.

Despite a higher numerical prevalence of steatosis in the follow-up group, no meaningful difference in liver stiffness was seen among the three groups: mean liver stiffness was 4.3 ± 1.3 kPa in CD_new, 4.5 ± 1.5 kPa in CD_GFD, and 4.9 ± 0.8 in non-CD controls. Although liver stiffness values did not differ meaningfully among the three groups, the observed burden of hepatic steatosis seen in GFD-treated CD warrants long-term follow-up.

### Exploratory Longitudinal Analysis

Paired baseline and follow-up assessments meeting the longitudinal paired-data criterion were available for 10 patients (Table 4). CAP increased from a median of 182.5 dB/m (IQR 172.5–188.0) at diagnosis to 217.0 dB/m (IQR 173.8–254.3) at follow-up (*p* = 0.047). None of the 10 patients had CAP ≥ 248 dB/m at diagnosis, compared with 3/10 (30.0%) at follow-up. Ultrasound-defined steatosis was present in 1/10 (10.0%) patients at diagnosis and 3/10 (30.0%) at follow-up (*p* = 0.625). Liver stiffness did not differ significantly between assessments (median 4.05 vs. 4.25 kPa, *p* = 0.334). Among these 10 patients with paired BMI measurements, BMI increased numerically from a median of 20.31 to 21.90 kg/m^2^ (*p* = 0.063). No significant paired differences were observed in fasting glucose, total cholesterol, or triglyceride concentrations. Regarding anti-tTG IgA measurements at diagnosis and follow-up, antibody levels decreased significantly (median 142.4 to 2.6 U/mL, *p* = 0.031).

## 4. Discussion

CD is widely recognized as a malabsorptive disease, with a general picture of underweight patients. However, one-third of patients are overweight or even obese at diagnosis [19], and, in the long term, weight changes can occur in either direction after diagnosis. Several reports have shown that GFD-treated CD patients have a greater metabolic burden, including increased prevalence of hepatic steatosis.

In the present cohort, in the longitudinal subset, CD patients evaluated after at least 2 years of GFD demonstrated higher median CAP at follow-up than at diagnosis; however, given the limited number of paired observations, this finding should be considered exploratory and hypothesis-generating, warranting validation in future studies. In the overall cross-sectional analysis, using a CAP threshold of ≥248 dB/m, hepatic steatosis was observed in 18.5% of newly diagnosed patients and 32.5% of patients at follow-up, indicating a numerically higher prevalence in long-term treated CD patients (prevalence ratio 1.76). This pattern aligns with previous reports describing the emergence of metabolic syndrome and MASLD during gluten-free diet adherence. Tortora et al. reported that while only 2% of patients fulfilled metabolic syndrome criteria at diagnosis, this increased to 29.5% after 12 months of GFD, similarly to Agarwal et al., who observed an increase from 14.3% at baseline to 29.5% after 1 year of diet [7,20]. Regarding hepatic steatosis, Ciccone et al. documented an increase from 1.6% at diagnosis to 11.0% during long-term follow-up, while Tovoli et al. reported non-alcoholic fatty liver disease (NAFLD) in 34.7% of celiac patients, including a higher prevalence among normal-weight individuals compared with controls (20% vs. 5.8%) [4,21]. Aligned with our findings, these data support the concept that metabolic and hepatic complications may emerge after initiation of a gluten-free diet in CD individuals, even in patients without traditional metabolic risk factors. On the other hand, others have shown that hepatic steatosis is more prevalent in pre-GFD CD patients than GFD-treated CD patients (50 vs. 35%), but the highest frequency of moderate and severe steatosis was seen in patients under GFD [22]. Variability in prevalence may also arise from differences in the definitions applied, with the adoption of the updated MASLD nomenclature more accurately reflecting the metabolic alterations observed in GFD-treated CD patients [23].

While steatosis prevalence in newly diagnosed CD in our cohort was lower than that reported in the overall population, the rate of steatotic liver disease (SLD) was numerically more prevalent in GFD-treated than in newly diagnosed CD patients, reaching comparable levels to those observed in non-CD controls and those reported in the general population [24] (Figure 1). Furthermore, there is significant variability in steatosis prevalence according to the assessment method reported [25,26,27], highlighting the importance of a multiparametric approach to liver evaluation, in order to improve identification of all patients at risk.

The development of MASLD in patients with CD is multifactorial and involves both disease-related and diet-related mechanisms [28,29]. Chronic low-grade intestinal inflammation and increased intestinal permeability in active CD, particularly in the setting of frequent contaminations or suboptimal dietary adherence, promote translocation of endotoxins and inflammatory mediators through the gut–liver axis, contributing to hepatic steatosis and liver injury. Also, the GFD, while essential for disease control, may inadvertently promote metabolic dysfunction due to its imbalanced nutritional composition, which is often characterized by higher fat and higher glycemic index. Along with the dietary changes, the social barriers encountered by CD individuals [30] can lead to more frequent physical inactivity and further contribute to weight gain, insulin resistance, and dyslipidemia, key drivers of hepatic fat accumulation. Not least, alterations in gut microbiota associated with both CD and GFD may further exacerbate metabolic disturbances and hepatic steatosis through disruption of the gut–liver axis. Taken together, these mechanisms explain why MASLD is an emerging metabolic complication in long-term GFD-treated CD individuals.

Although expected to improve metabolic markers and aid in weight loss, GFD initiation was actually demonstrated to exacerbate existing overweight and obesity and contribute to liver steatosis. A potential explanation proposed for this is not the elimination of gluten, but rather the poor dietary choices that CD patients face in the absence of adequate nutritional counselling. Previous observational data have shown that the increased consumption of packaged gluten-free foods was significantly related to NAFLD. Moreover, specific GFD dietary habits, like the consumption of sweet convenience foods, were correlated with the degree of risk of NAFLD [31].

Frustration and social constraints may be reasons people choose gluten-free versions of popular foods like cookies and cakes, pizza, pasta, sandwiches, and burgers, which are highly processed to improve structure and palatability. In general, processed gluten-free products have unfavorable nutritional profiles compared to their gluten-containing counterparts, with higher contents of simple carbohydrates, sugars and saturated fats, while also being lower in fiber. To replace the structure and taste of gluten, commercial gluten-free products contain mostly refined starches with high glycemic indices (rice, corn, potato, tapioca), which trigger insulin spikes and promote liver fat storage. Moreover, gluten-free processed grain products (e.g., breads, cereals, and crackers) are often lower in B vitamins, iron, zinc, magnesium, and potassium [32].

CD is associated with significant intestinal microbiota alteration, characterized by an increased abundance of potentially pathogenic gram-negative bacteria, such as *Proteobacteria*, which promote inflammation, alongside a reduced prevalence of *Bifidobacterium*, *Lactobacillus*, and butyrate-producing bacterial species. Overall microbial diversity is diminished, contributing to mucosal inflammation and increased intestinal permeability [33]. A strong association between intestinal dysbiosis and celiac disease has been consistently demonstrated, and current research is focused on clarifying whether celiac disease predisposes to dysbiosis or whether dysbiosis itself contributes to the development of celiac disease. A longitudinal study investigating intestinal dysbiosis markers before CD onset in children found that proinflammatory bacteria were increased, while butyrate-producing bacteria like *Faecalibacterium prausnitzii* and *Clostridium clostridioforme* were decreased, indicating insufficient butyrate production in these patients [34]. Moreover, cross-feeding interactions between *Bifidobacterium* species and butyrate-producing bacteria, including *Faecalibacterium prausnitzii*, *Anaerostipes*, *Eubacterium*, and *Roseburia* species, have been shown to enhance butyrate production, whereas reduced levels of *Bifidobacterium* are associated with increased intestinal populations of pro-inflammatory cells. Although *Bifidobacterium* does not directly produce butyrate, it ferments dietary prebiotic fibres, such as inulin-type fructans and arabinoxylans, generating acetate and lactate as metabolic byproducts. These metabolites are subsequently utilized by butyrate-producing bacteria to synthesize butyrate, a key short-chain fatty acid essential for intestinal and metabolic homeostasis [35]. Butyrate plays a pivotal role in the gut–liver axis, and emerging evidence suggests that it may contribute to the management of MASLD by reducing hepatic inflammation, modulating genes involved in lipid and glucose metabolism, decreasing hepatic fat accumulation, and regulating triglyceride levels [36]. The gut microbiota of CD patients on a strict GFD rarely mirrors that of healthy individuals. Some studies indicate that populations of beneficial *Bifidobacterium* can actually decline further due to the elimination of certain fibers often found in gluten-containing grains [37]. Even after 2 years of GFD, beneficial bacteria remain decreased in some cases [38], while in healthy individuals, GFD may reduce microbial diversity. GFD itself may alter the gut microbiota by excluding specific prebiotic carbohydrates that are essential for sustaining beneficial bacterial populations, thereby contributing to persistent compositional imbalances [39]. A recent study confirmed that 1 year of GFD did not reverse abnormalities and had a negative impact on the intestinal microbiome, with decrease of bifidobacterial abundance, increase in proteolytic species, and a reduction in arabinoxylan-degrading families [40]. Recent research found that CD patients, both newly diagnosed and those on long-term GFD, lack key microbial families responsible for breaking down dietary fiber, such as *Prevotella* spp. Colonization of germ-free mice with *Prevotella* spp. resulted in increased production of short-chain fatty acids. Furthermore, in gluten-sensitized mice expressing the celiac disease susceptibility gene HLA-DQ8, an inulin-supplemented diet enhanced microbial saccharolytic activity, promoted short-chain fatty acid production, and accelerated small-intestinal mucosal healing during adherence to GFD [41].

While CD patients are traditionally considered malnourished prior to diagnosis, emerging evidence suggests that nutritional status may, in some cases, further deteriorate following diagnosis. Although weight gain after initiation of a GFD may be misinterpreted as a beneficial response reflecting improved nutrient absorption, it should be monitored cautiously. Whereas lean malnourished individuals experience deficiencies in both energy intake and essential nutrients, obese malnourished individuals consume excess calories while remaining deficient in key micronutrients, resulting in a severe “double burden” of disease. Similarly, the resolution of digestive symptoms following a GFD initiation does not necessarily indicate complete restoration of intestinal health, as alterations in the gut microbiome may still persist despite clinical improvement.

Our findings add to the current evidence that long-term treated CD patients have a greater metabolic and hepatic steatosis burden. The clinical implications stand in recommending routine metabolic work-up as part of CD follow-up and enforcing nutritional counselling in at-risk patients. Further research should look at the therapeutic management of metabolic complications in GFD-treated CD patients, as well as implementing strategies to improve the suboptimal nutritional profile of many processed gluten-free products.

Recent guidelines for CD follow-up recognize the importance of monitoring metabolic dysfunction and liver involvement over time and recommend regular evaluation of metabolic risk factors, including serum glucose, lipid profile, and liver enzymes [42,43]. However, because MASLD may be present even in patients with normal aminotransferase levels [44], reliance on biochemical testing alone may fail to identify a proportion of affected individuals. In light of the increasing prevalence of SLD in long-term treated CD patients, the incorporation of imaging-based liver assessment methods, such as ultrasound and controlled attenuation parameter (CAP) evaluation, may improve the detection of SLD during follow-up of patients.

Despite the relatively small sample size from a single center, the absence of structured dietary intake data of participants, also lack of standardized quantitative assessment of alcohol consumption, which represents significant limitations, our study has several important strengths. First, the analysis of GFD-treated CD patients in comparison with newly diagnosed CD in the subgroup of longitudinally assessed patients provides a clinically meaningful framework for evaluating metabolic changes associated with the initiation of a GFD. This subset analysis enabled an exploratory assessment of the metabolic trajectory from diagnosis through follow-up within the spectrum of CD. However, the primary component of the study was cross-sectional, which cannot establish temporal or causal effects of GFD. Second, liver steatosis was assessed using quantitative methods, specifically the CAP obtained by transient elastography, which offers an objective and reproducible measurement compared with conventional ultrasound alone. The combined use of imaging modalities strengthens the validity of steatosis detection. However, excluding patients with diabetes and those receiving glucose- or lipid-lowering therapies may have selected a metabolically healthier population, thereby limiting generalizability and potentially underestimating the real-world burden of metabolic liver disease among CD individuals. The lack of operator blinding in ultrasound and transient elastography examinations is also a limitation of our work. Not least, the limited sample size reduced statistical precision and may have impaired the detection of modest between-group differences, with a consequent risk of type II error.

## 5. Conclusions

Long-term GFD-treated patients showed higher BMI and numerically higher imaging-defined hepatic steatosis prevalence than newly diagnosed CD patients, although the primary cross-sectional differences in steatosis were not statistically significant. In the exploratory paired longitudinal subset, median CAP was higher at follow-up than at diagnosis, suggesting a risk of metabolic steatopathy in long-term GFD-treated patients, but this finding requires confirmation in larger prospective cohorts. Further studies with adequate adjustment for adiposity, dietary factors, and other potential confounders are needed to better define metabolic and hepatic changes during long-term CD follow-up. Our findings reinforce the importance of metabolic monitoring and integration of nutritional educational interventions into long-term CD management.

## Figures and Tables

**Figure 1:**
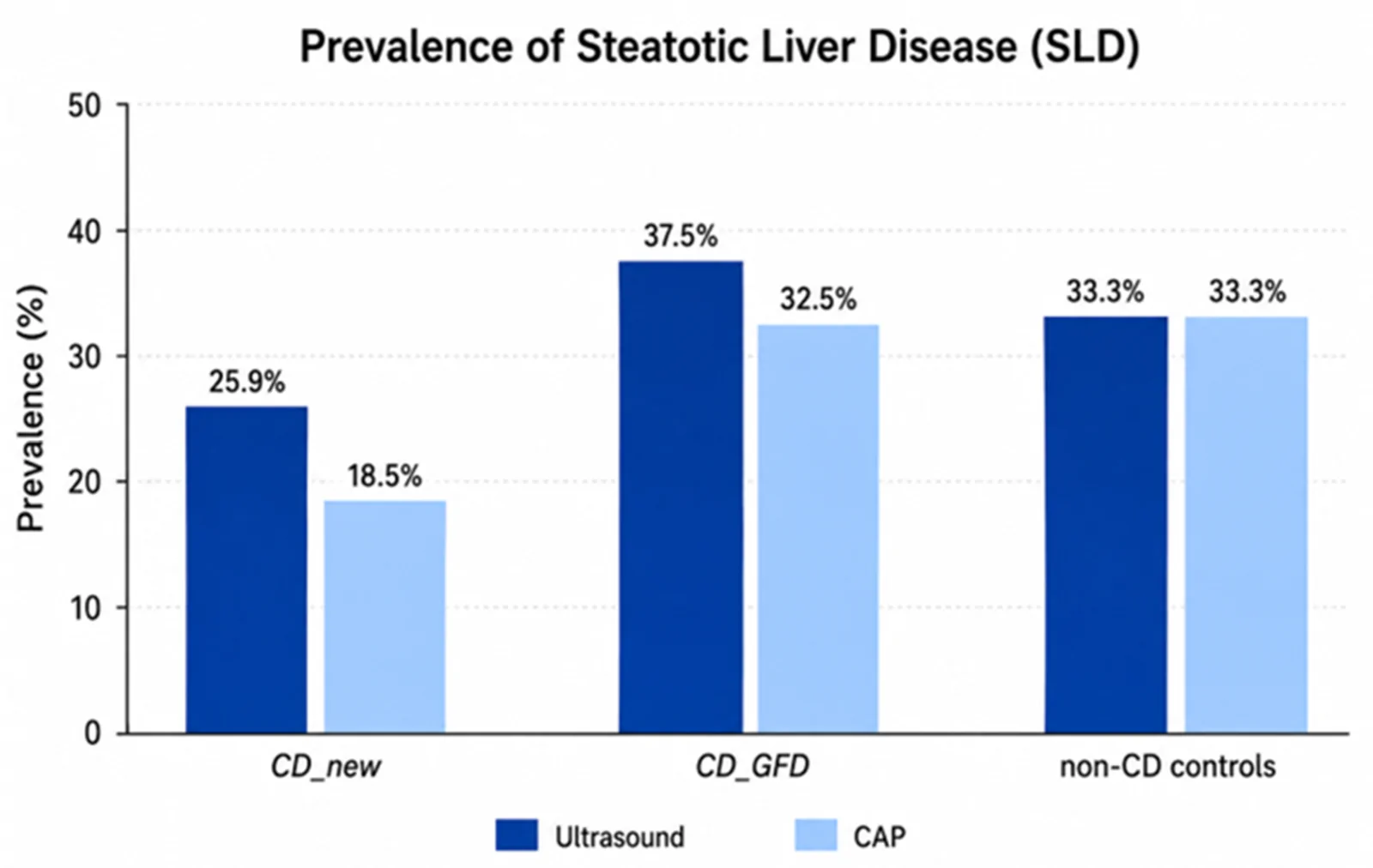
Numerical prevalence of SLD in newly diagnosed CD, GFD-treated CD, and non-CD controls. (Between-group comparison *p* = 0.687 for ultrasound-defined steatosis and *p* = 0.381 for CAP-defined steatosis).

**Table 1:** Demographic, serological, and histopathological characteristics of study participants.

	CD_New (*n* = 27)	CD_GFD (*n* = 40)	Non-CD Controls (*n* = 18)
Age, years (median, IQR)	40 (33.5–47)	42 (33.3–53)	46 (38–62.8)
Female sex, *n* (%)	24 (88.9)	32 (80.0)	13 (72.2)
BMI, kg/m^2^ (mean ± SD)	21.9 ± 3.3	24.5 ± 5.4	23.1 ± 4.4
GFD-duration, years (median, IQR), range	-	6 (2.8–8), range 2–21	-
Marsh-Oberhuber grade ≥ 3, *n* (%)	27 (100)	4 (10)	0 (0)
tTG IgA, U (median, IQR)	134.3 (46.5–200)	4.6 (2.1–14.3)	3.1 (1.1–4.1)

**Table 2:** Lipid profile comparison between newly diagnosed and GFD-treated CD patients.

Lipid Parameter	CD_New	CD_GFD	*p*-Value
Total cholesterol, mg/dL	172.9 ± 43.5	183.1 ± 42.9	0.358
Triglycerides, mg/dL	84.1 ± 32.5	79.8 ± 32.5	0.605
HDL-C, mg/dL	57.3 ± 17.7	60.1 ± 17.4	0.637
LDL-C, mg/dL	109.5 ± 35.7	107.1 ± 36.8	0.830

**Table 3:** Hepatic steatosis comparison as assessed by ultrasound and CAP between newly diagnosed, GFD-treated CD patients and non-CD controls.

Liver Steatosis	CD_New	CD_GFD	Non-CD Controls	*p*-Value
Ultrasound-defined steatosis, *n*/total (%)	7/27 (25.9)	15/40 (37.5)	6/18 (33.3)	0.687
CAP-based steatosis, *n*/total (%)	5/27 (18.5)	13/40 (32.5)	6/18 (33.3)	0.381
Mean CAP, dB/m	204.1 ± 37.2	217.0 ± 75.4	232.3 ± 41.6	0.295

**Table 4:** Longitudinal analysis of metabolic parameters, hepatic steatosis, and liver stiffness among paired newly diagnosed and GFD-treated CD patients.

Variable	At Diagnosis	Follow-Up	*p*-Value
CAP, dB/m	182.5 [172.5–188.0]	217.0 [173.8–254.3]	0.047
CAP ≥ 248 dB/m	0/10 (0%)	3/10 (30.0%)	0.250
CAP ≥ 268 dB/m	0/10 (0%)	2/10 (20.0%)	0.500
CAP ≥ 285 dB/m	0/10 (0%)	1/10 (10.0%)	1.000
Ultrasound steatosis	1/10 (10.0%)	3/10 (30.0%)	0.625
Liver stiffness, kPa	4.05 (3.30–4.55)	4.25 (3.88–5.03)	0.334
BMI, kg/m^2^	20.31 (18.72–22.01)	21.90 (19.90–23.52)	0.063
Fasting glucose, mg/dL	78 (75–86)	82 (78–84)	0.734
Total cholesterol, mg/dL	139.5 (130.8–182.8)	159.0 (141.5–172.5)	0.910
Triglycerides, mg/dL	69.5 (59.0–87.8)	65.5 (48.0–87.3)	0.576

## Data Availability

Data are available upon request from the corresponding author.

## Abbreviations

The following abbreviations are used in this manuscript:

CD	Celiac disease
GFD	Gluten-free diet
MASLD	Metabolic dysfunction-associated steatotic liver disease
NAFLD	Non-alcoholic fatty liver disease
SLD	Steatotic liver disease
CAP	Controlled attenuation parameter
